# Genetically engineered bananas resistant to Xanthomonas wilt disease and nematodes

**DOI:** 10.1002/fes3.101

**Published:** 2017-03-29

**Authors:** Leena Tripathi, Howard Atkinson, Hugh Roderick, Jerome Kubiriba, Jaindra N. Tripathi

**Affiliations:** ^1^ International Institute of Tropical AgricultureNairobiKenya; ^2^ Centre for Plant SciencesUniversity of LeedsLeedsUK; ^3^ National Agricultural Research LaboratoriesPO Box 7084KampalaUganda

**Keywords:** Banana, improvement, genetic engineering, Xanthomonas wilt, nematode, Africa

## Abstract

Banana is an important staple food crop feeding more than 100 million Africans, but is subject to severe productivity constraints due to a range of pests and diseases. Banana Xanthomonas wilt caused by *Xanthomonas campestris* pv. *musacearum* is capable of entirely destroying a plantation while nematodes can cause losses up to 50% and increase susceptibility to other pests and diseases. Development of improved varieties of banana is fundamental in order to tackle these challenges. However, the sterile nature of the crop and the lack of resistance in *Musa* germplasm make improvement by traditional breeding techniques either impossible or extremely slow. Recent developments using genetic engineering have begun to address these problems. Transgenic banana expressing sweet pepper *Hrap* and *Pflp* genes have demonstrated complete resistance against *X. campestris* pv. *musacearum* in the field. Transgenic plantains expressing a cysteine proteinase inhibitors and/or synthetic peptide showed enhanced resistance to a mixed species population of nematodes in the field. Here, we review the genetic engineering technologies which have potential to improve agriculture and food security in Africa.

## Introduction

Closing the yield gap of staple crops is a priority for ensuring future food security, especially in developing nations (Godfray et al. 2010). The population of Africa is projected to double between 2015 and 2050 to 2.5 billion and increase further to 4.4 billion by 2100 by which time 38% of the global population is projected to be African (UN 2015). In many parts of the world emphasis can be placed on cereals to address the demands of population growth (West et al. 2014) but not in some key areas in Sub‐Saharan Africa (SSA).

Banana including plantain (*Musa* spp.) is an important staple crop in tropics. Annual global production of banana is about 145 million tons (FAOSTAT 2014). Approximately a third of that production is in Africa, and Africa accounts for about 72% of production of plantains (FAOSTAT 2014). Investment in banana improvement holds great potential for improving food security as these crops feed more people per unit area of production than other staple crops (West et al. 2014). For instance, Uganda produces 30% of the global production of cooking bananas and has the highest consumption per capita (FAOSTAT 2014). In southeastern Nigeria, smallholder farmers generate up to 30% of their income from plantain cultivation (Pasberg‐Gauhl and Gauhl 1996). In Central and West Africa, plantains account for about 32% of total *Musa* production (Lescot 2008), which feed approximately 70 million people with >25% of their carbohydrates and 10% of their food energy (Ortiz and Vuylsteke 1996; Robinson 1996).

Most cultivated banana varieties are triploids with low to no fertility generated by hybridizations between two diploid species, *Musa acuminata* and *M. balbisiana*, which contribute to the A and B genomes, respectively (Ortiz et al. 1995). The sweet dessert banana that forms the bulk of the export market and East African highland bananas (EAHB) are AAA, plantains and East African dessert bananas are AAB, and most other cooking bananas are ABB (Simmonds 1987).

Banana production is severely hampered by several pests and diseases, particularly on low‐input, subsistence farms. Banana Xanthomonas wilt (BXW) caused by *Xanthomonas campestris* pv. *musacearum* is seriously threatening the banana production in East Africa (Tripathi et al. 2009; Shimwela et al. 2016a). The disease starts with wilting of leaves or male bud and premature ripening of fruits leading to death of plant and rotting of fruits. Where it occurs, BXW causes acute infections that can lead to a complete loss of a plantation. It caused 30–50% decrease in banana yields in Uganda between 2001 and 2004 (Karamura et al. 2006; Shimwela et al. 2016a). Economic losses of about $2–8 billion have been reported over a decade in the East Africa (Tripathi et al. 2009; Nkuba et al. 2015; Shimwela et al. 2016a). BXW disease is transmitted mainly by insects, contaminated farming tools, infected planting materials, and probably rain splash (Shimwela et al. 2016a,b). It can be contained by the use of cultural practices such as removal of the male bud to prevent insect transmitted infection, using sterilized farming tools, destroying infected plants, and using clean pathogen‐free planting materials. However, the adoption of these practices is inconsistent as these techniques are labor intensive and may enhance disease spread if cutting of plants occurs during rainy season (Shimwela et al. 2016a,b). The disease affects all banana varieties and no resistant source has been identified in *Musa* germplasm yet.

Nematodes cause losses globally to banana production. Analysis of data from experimental applications of nematicides across a range of African countries has demonstrated yield responses of 71 ± 16% over 3 years after nematicide application (Atkinson 2003). Losses of >50% have been confirmed in a field trial with plantain (Roderick et al. 2012a). Nematodes are often controlled in commercial banana plantations by periodic application of environmentally damaging pesticides, but they are not normally available or suitable for smallholders in Africa. Crop rotation is not often possible for such farmers, many of whom have insufficient land to accept the associated yield loss, given that plantains out produce all other staple crops in conditions that favor them.

Development of nematodes or banana Xanthomonas wilt*‐*resistant cultivars by traditional crosspollination techniques is hampered by the sterility of the polyploid genomes of cultivated banana and plantains (Lorenzen et al. 2010). However, conventional breeding has produced hybrids with resistance to *Radopholus similis* though these tend to remain moderate hosts for *Pratylenchus* species (Quénéhervé et al. 2009). No hybrid has shown resistance to the concurrent infections by several nematode species (Pinochet 1988; De Waele and Elsen 2002; Lorenzen et al. 2010) as required to manage them on banana and plantain crops.

No resistant varieties of banana have been identified with both nematode and Xanthomonas wilt resistance, but transgenic plants with both of these resistance traits have been valued for Uganda alone at $962 m over a 30‐year period (Kalyebara et al. 2007). Male and female sterility of most edible cultivars, lack of crossfertile wild relatives, and clonal propagation of banana all contribute to no risk of gene flow from transgenic banana plants to either wild or cultivated plants. Deployment of farmer preferred transgenic cultivars is unlikely to adversely affect the already very low genetic variability in the banana crop due to its perennial nature and a reliance on very few cultivars across large geographical areas. This review describes progress on developing transgenic banana resistance to both Xanthomonas wilt disease and nematodes and key issues to be resolved before their deployment to growers in Africa.

## Xanthomonas wilt Resistant Banana

Genetic engineering is an important tool that facilitates transfer of genes for useful agronomic traits across species. It can complement conventional breeding of banana by allowing the bottlenecks of breeding for developing improved varieties to be overcome. In the absence of known host plant resistance among banana genotypes, genetic engineering provides a cost‐effective alternative technique to develop Xanthomonas wilt resistant banana varieties. Host plant resistance against pathogens can be enhanced by expressing resistance (R) genes, antimicrobial genes, or defense genes (Tripathi et al. 2016; Table 1). The Hypersensitive Response Assisting Protein *(Hrap)* and Plant Ferredoxin Like Protein (*Pflp)* genes from sweet pepper (*Capsicum annuum*) are defense genes which can intensify the hypersensitive response (Lin et al. 1997; Chen et al. 2000). These genes have provided resistance against various bacterial pathogens such as *Erwinia, Pseudomonas, Ralstonia*, and *Xanthomonas* spp. in transgenic *Arabidopsis,* tobacco, tomato, orchids, calla lily, and rice (Tang et al. 2001; Ger et al. 2002; Liau et al. 2003; Huang et al. 2004, 2007; Pandey et al. 2005; Yip et al. 2007).

**Table 1:** List of genes introduced to various crops for developing resistance to bacterial disease and nematodes

Resistance Technology/Target Gene	Origin	Target Organism	Crop	Mode of Action	Resistance		References
					Green house	Field	
*Bacterial Disease Resistance*							
*Hrap*	Sweet pepper	*X. campestris pv. musacearum*	Banana	Hypersensitivity Response	Full	Full	Tripathi et al. (2010, 2014a)
*Pflp*	Sweet pepper	*X. campestris pv. musacearum*	Banana	Hypersensitivity Response	Full	Full	Namukwaya et al. (2012); Tripathi et al. (2014a)
*Xa21*	Rice	*X. campestris pv. musacearum*	Banana	Pathogen Recognition	Full	–	Tripathi et al. (2014b)
*Pto*	Tomato	*X. campestris pv. vesicatoria*	Tomato	Resistance (R) Gene	Enhanced	–	Tang et al. (1999)
*Bs2*	Sweet pepper	*X. campestris pv. vesicatoria*	Tomato	Resistance (R) Gene	Enhanced	–	Tai et al. (1999)
*Rxo1*	Maize	*X. oryzae pv. oryzicola*	Rice	Resistance (R) Gene	Enhanced	–	Zhao et al. (2005)
*Npr1*	Arabidopsis	*X. oryzae pv. oryzae*	Rice	Systemic Acquired Resistance	Enhanced	–	Chern et al. (2005)
*NH1*	Rice	*X. oryzae pv. oryzae*	Rice	Systemic Acquired Resistance	Enhanced	–	Yuan et al. (2007)
*EFR*	Arabidopsis	*Ralstonia solanacearum, Xanthomonas perforans*	Tomato	Pathogen Recognition	Enhanced	–	Lacombe et al. (2010)
D4E1	Synthetic	*X. populi pv. populi*	Popular	Cecropin Antimicrobial Peptide	Enhanced	–	Mentag et al. (2003)
*Nematode Resistance*							
*CC*II	Maize	*R. similis, H. multicinctus, Meloidogyne* sp.	Plantain	Antifeedant	84%	98%	Roderick et al. (2012b); Tripathi et al. (2015)
Peptide	Synthetic	*R. similis, H. multicinctus, Meloidogyne* sp.	Plantain	Behavioral Repellent	66%	99%	Roderick et al. (2012b); Tripathi et al. (2015)
*CC*II + Peptide	Synthetic	*R. similis, H. multicinctus, Meloidogyne* sp.	Plantain	As above	70%	95%	Roderick et al. (2012b); Tripathi et al. (2015)
OcIΔD86	Rice	*R. similis*	Banana	Antifeedant	70%	–	Atkinson et al. (2004a)
*Cry5B*	*B. thuringiensis*	*M. incognita*	Tomato	Bt Toxin	64%	–	Li et al. (2008)
*16D10*	*M. incognita*	*M. incognit, M. Javanica, M. arenaria, M. hapla*	*Arabidopsis*	RNAi	93%	–	Huang et al. (2006)
*tp*	*M. incognita*	*M. incognita*	Soybean	RNAi	82%	–	Ibrahim et al. (2010)
*msp*	*M. incognita*	*M. incognita*	Soybean	RNAi	85%	–	Ibrahim et al. (2010)
*cb‐1*	*R. similis*	*R. similis*	Tobacco	RNAi	73%	–	Li et al. (2015a)
*crt*	*R. similis*	*R. similis*	Tomato	RNAi	75%	–	Li et al. (2015b)
Splicing Factor	*M. incognita*	*M. incognita*	Tobacco	RNAi	100%	–	Yadav et al. (2006)
Integrase	*M. incognita*	*M. incognita*	Tobacco	RNAi	99%	–	Yadav et al. (2006)
*flp‐14*	*M. incognita*	*M. incognita*	Tobacco	RNAi	50%	–	Papolu et al. (2013)
*flp‐18*	*M. incognita*	*M. incognita*	Tobacco	RNAi	58%	–	Papolu et al. (2013)

Full – transgenic lines identified with full resistance to bacterial pathogen, and Enhanced – transgenic lines identified with reduced disease symptoms. Best line percentage resistance to nematodes calculated from nematodes/100 g root relative to infected nontransgenic control plants.

Transgenic bananas have been generated by inserting *Hrap* or *Pflp* gene in embryogenic cell suspensions of banana cultivars, the AAB sweet banana cultivar ‘Sukali Ndiizi’, and the AAA‐EAHB cultivar ‘Nakinyika’, through *Agrobacterium‐*mediated transformation (Tripathi et al. 2010; Namukwaya et al. 2012). The transgenic events were analyzed to confirm the presence of transgene by PCR and integration of transgene in banana genome by Southern blot analysis. Several of these transgenic events showed enhanced resistance under laboratory and glasshouse conditions (Tripathi et al. 2010; Namukwaya et al. 2012). The promising transgenic events showing 100% resistance against *Xcm* in glasshouse experiments were selected for confined field trial. Sixty‐five transgenic events (40 *Hrap* gene lines and 25 *Pflp* gene lines) were evaluated for disease resistance in a confined field trial at the National Agricultural Research Laboratory (NARL), Kawanda, Uganda. Complete resistance to Xanthomonas wilt disease was demonstrated for 11 transgenic events (7 *Hrap* lines and 4 *Pflp* lines) for both mother and progeny crops (Tripathi et al. 2014a). Control nontransgenic plants developed disease symptoms and wilted completely. The results from field trial experiment confirmed the transfer of the disease resistance trait from mother to progeny. These 11 transgenic events, besides showing absolute resistance to Xanthomonas wilt disease, also showed agronomic characteristics (flowering and yield) similar to nontransgenic control varieties (Tripathi et al. 2014a). These transgenic events were further evaluated in a second confined trial to measure agronomic performance. As bacterial pathogens evolve fast, there is risk of breaking down of resistance in transgenic plants developed using single gene. To avoid or delay this situation, we are developing transgenic banana varieties using stacked genes (*Hrap‐Pflp*). The transgenic banana expressing stacked *Hrap* and *Pflp* genes did not show higher or additive resistance against pathogen in comparison to individual genes; however, stacking might provide the benefit of durable resistance in case one transgene function is lost (Muwonge et al. 2016).

The rice pattern recognition receptor (PRR) XA21 has also been tested for resistance against *X. campestris pv. musacearum* in order to identify additional disease resistance genes for use in gene pyramiding strategies. The transgenic rice overexpressing *Xa21* gene confers resistance to the bacterial pathogen *X. oryzae* pv. *oryzae* (Ronald et al. 1992; Wang et al. 1996). Transgenic banana expressing rice *Xa21* gene were developed and tested for Xanthomonas wilt disease resistance. These transgenic plants demonstrated enhanced resistance against *X. campestris pv. musacearum* under glass house conditions (Tripathi et al. 2014b).

Several other potential transgenes have been identified that suppress development of disease lesions in other plants species or shown in vitro antibacterial effects against *Xanthamonas* sp. Transgenic tomato expressing the R‐genes, *Pto* or *Bs*2, showed resistance against *X. campestris pv. vesicatoria* (Tai et al. 1999; Tang et al. 1999). The maize *Rxo*1 gene provides resistance against *X. oryzae pv. oryzicola* causing bacterial streak disease in rice (Zhao et al. 2005). Overexpression of Arabidopsis *NPR1* or the rice *NH1* gene enhanced resistance to the rice bacterial blight pathogen *X. oryzae pv. oryzae* (Chern et al. 2005; Yuan et al. 2007). Expression of receptor EFR from *Arabidopsis thaliana* confers resistance against range of phytopathogenic bacteria in *Nicotiana benthamiana,* tomato, rice, and wheat (Lacombe et al. 2010; Schoobeek et al. 2015; Schwessinger et al. 2015). Cecropins derived from the Cecropia moth (*Hyalophora cecropia*) including native (cecropin B), synthetic (Shiva‐1, D4E1), and mutant (SB‐37, MB39) have shown antimicrobial activity against bacterial pathogens *X. campestris* and *X. populi* (Nordeen et al. 1992; Kaduno‐Okuda et al. 1995; Rajasekaran et al. 2001; Mentag et al. 2003).

## Nematode Resistant Bananas

The key nematode pests of banana in SSA are the migratory species *Radopholus similis*,* Pratylenchus goodeyi*,* P. coffeae*,* Helicotylenchus multicinctus,* and sedentary *Meloidogyne* spp. The migratory endoparasite *R. similis* is considered the most damaging where it occurs causing extensive root necrosis as the nematode migrates through the root feeding. This reduces root function and compromises plant anchorage leading to toppling during storms. *Pratylenchus* spp. are becoming increasingly prevalent pests of *Musa* across Africa, especially on plantain in West Africa, resulting in growing concern for their potential impact (Coyne 2009). They impose root pathology similar to *R. similis* (Bridge et al. 1997). *H. multicinctus* occurs in almost all banana‐growing areas mainly in root cortex causing some necrosis. The sedentary root parasite *Meloidogyne* spp. differs in modifying plant cells into a feeding site at one locale (Gowen et al. 2005). Infestations of complexes of species are prevalent and the combination of nematode species present in banana plantations varies with the locality (Coyne et al. 2013).

Several transgenic defenses against nematodes are in different stages of development (Table 1). Cysteine proteinases are major digestive enzymes of many nematodes and can be inhibited by cysteine proteinase inhibitors (cystatins). The expression of plant cystatins by roots suppresses nematode growth and reproduction on several plants in containment including tomato (Urwin et al. 1995), *Arabidopsis* (Urwin et al. 1997, 2000), rice (Vain et al. 1998), dessert banana (Atkinson et al. 2004a), aubergine (Papolu et al. 2016), and Easter Lily (Vieira et al. 2015). High levels of efficacy have also been established in confined field trials for both potato expressing an engineered rice grain cystatin (Urwin et al. 2001, 2003; Lilley et al. 2004) and plantain expressing a maize kernel cystatin (Roderick et al. 2012b).

**Figure 1:**
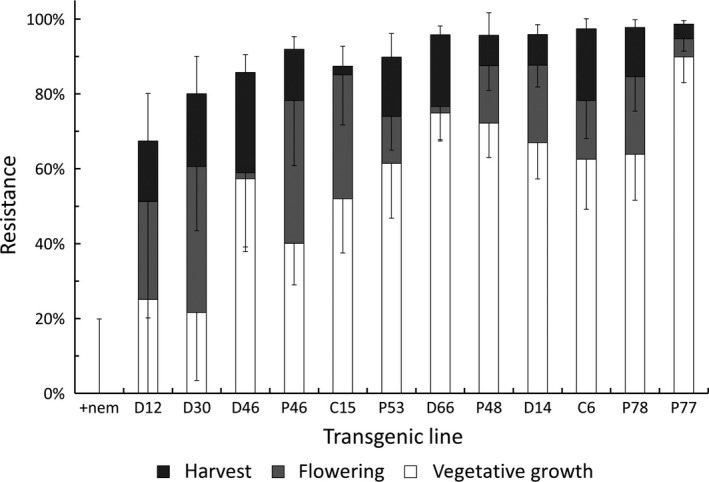
Stacked columns of cumulative percentage resistance (mean ± SEM) for the periods vegetative growth, flowering, and harvest (i.e., Line D30 had 22% resistance at vegetative sampling, 61% resistance at flowering sampling, and 80% resistance at harvest flowering) for 12 transgenic lines relative to the control plants to which nematodes were added before planting (+nem). Data are based on Tripathi et al. 2015. The expressed transgenes in the independent, transgenic events are as follow: C, cystatin; P, peptide; and D, both C and P.

A second well‐developed transgenic resistance defense is based on expression of peptides that reduce invasion of roots without being lethal to nematodes. The peptides undergo retrograde transport along certain chemosensory dendrites to neuronal cell bodies of several nematodes including *R. similis* resulting in a loss of orientation to roots (Winter et al. 2002; Wang et al. 2011; Roderick et al. 2012b). The peptide that has been deployed in plantains is a disulfide‐constrained 7‐mer with the amino sequence CTTMHPRLC (Winter et al. 2002; Roderick et al. 2012b). It has provided resistance to *Globodera pallida* in the glasshouse (Lilley et al. 2011) and field (Green et al. 2012). Both the peptide and a cystatin provided a high level of resistance in plantain to both *R. similis* and *H. multicinctus* with evidence of an accumulative benefit as the crop advanced to harvest as the introduced nematodes failed to maintain their density on the growing root system (Tripathi et al. 2015; Fig. 1).

*Bacillus thuringiensis* derived Bt endotoxin genes, similar to the highly effective insecticidal genes deployed in several crops, have also demonstrated an ability to suppress *Meloidogyne* species (Li et al. 2008; Zhang et al. 2012; Yu et al. 2015). Plant lectins have also been shown to suppress *M. incognita* but are toxic to insects and mammals (Burrows and de Waele 1997). RNA interference (RNAi) based defenses are being developed and have shown promise against *Meloidogyne* species (Huang et al. 2006; Yadav et al. 2006; Ibrahim et al. 2011; Papolu et al. 2013), *R. similis* (Haegeman et al. 2009; Li et al. 2015a,b), and *Pratylenchus* species (Joseph et al. 2012; Tan et al. 2013). However, none of these resistance technologies have been deployed into banana as they lack the broad control required for concomitant infections typical in banana plantations (Wei et al. 2003).

## Food and Environmental Biosafety

Bioinformatics approaches comparing the amino acid sequences of PFLP and HRAP proteins to known allergens (AllergenOnline.org and NCBI) and toxins (NCBI) confirmed that both the proteins are safe for human consumptions and do not have similarity with any toxin or allergen in database. The transgenic banana expressing *Hrap* or *Pflp* gene will be evaluated for food and environmental safety during next field trial.

There is a well‐established case for the food and environmental safety of both the cystatin and peptide defenses. The rice and maize seed cystatins deployed in banana are not novel dietary proteins. They are consumed as part of the staple diet of many Africans and a similar protein is present in human saliva (Veerman et al. 1996). Experimental approaches concluded that they are neither toxins (Atkinson et al. 2004b) nor allergens (MAFF UK 2000). They also share no similarity with any known toxic or allergenic proteins in databases. The peptide expressed in plantains is destroyed by cooking and by simulated intestinal fluid. It is not recognized as a potential allergen by Allergenonline (http://www.allergenonline.com) or Allermatch (http://allermatch.org; Fiers et al. 2004), two tools that meet Food and Agriculture Organization/World Health Organization (FAO/WHO) Codex alimentarius guidelines for allergenicity assessment (http://bit.ly/CodexAlimentarius). The lack of allergenicity of the 1.16‐kDa peptide is also consistent with the observation that proteins of less than 3 kDa do not normally elicit an allergic response in mammals (Van Beresteijn et al. 1994).

Expression of the engineered rice cystatin by potato plants does not pose a measurable environmental risk to aerial invertebrate associates of a transgenic potato crop (Cowgill et al. 2002a, 2004; Cowgill and Atkinson 2003), or perturb soil organism communities in the field (Cowgill et al. 2002b). Free‐living soil nematodes are also unaffected by potato plants expressing the engineered rice cystatin (Green et al. 2012). The peptide is rapidly degraded in the soil, presumably being utilized by soil microorganisms. It is not lethal to nontarget invertebrates at levels above those produced by transgenic plants (Wang 2009). Its release from transgenic potato does not perturb nontarget communities of soil nematodes in the field (Green et al. 2012). Both food and environmental biosafety can be enhanced further by controlling expression under promoters that express preferentially in roots (Green et al. 2012). This strategy may also enhance the effectiveness of the peptide defense. Placing the peptide under control of a root‐cap‐specific promoter provided 94.9 ± 0.8% resistance to *G. pallida* in contained potato plant trial compared to 34.4 ± 8.4% resistance when the peptide was under control of the constitutive CaMV35S promoter (Lilley et al. 2011).

## Future Perspectives

The progress of innovations to the market has been charted for a wide range of technologies using the Hype cycle (Fenn and Raskino 2008). Crucial to applying this approach is defining a metric of visibility. In the case of transgenic technologies citation number based on keyword searches, as used in a meta‐analysis of the agronomic and economic impacts of GM crops (Klümper and Qaim 2014), provide a useful measure of the developmental state of specific genetic modifications. Citation frequency falls as workers with fundamental science interests disengage from the field leaving only those involved in translational and subsequent research for those applications showing commercial potential. Additionally, the years for citations to accumulate are less for more recent, translational research than the older publications on which that effort is based.

**Figure 2:**
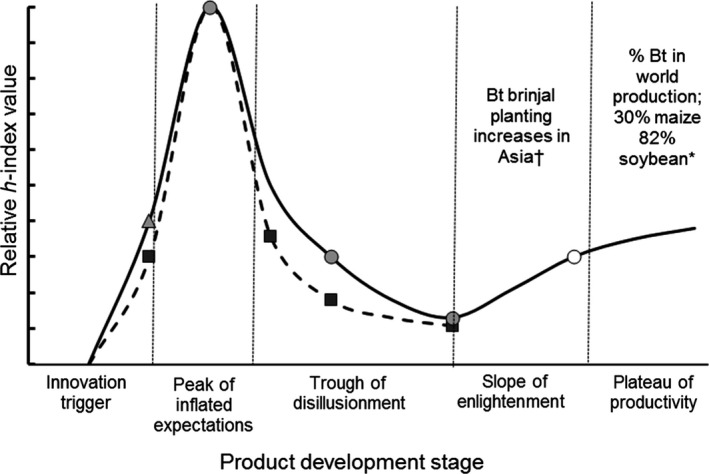
Gartner Hype cycle applied to development of Bt technology for transgenic insect control (solid line) and nematode resistance (dashed line and squares) expressed as percentage of the highest citation value in the peak of inflated expectations. The search terms used for Bt technology were “Bt + insect + crop” followed by stepped addition of “field”, then “yield”, then “benefit”, and finally “Bt grower + society”. For nematode resistance, these were “nematode + transgenic” followed by stepped addition of “crop”, then “field”, then “yield”, and finally “improved”. Estimated time to plateau of productivity for Bt technology; light‐gray triangle, >10 years; gray circle <5–10 years; and open circles <2 years or on plateau. †, Herring 2015; *, James 2014.

The anti‐insect protein from *Bacillus thuringiensis* (Bt) that confers resistance to certain insects can act as a comparator for nematode resistance technologies when applying the Hype cycle method (Fig. 2). Bt represents a mature resistance technology in food crops that has a stable market based on real benefits. A limitation of fitting the Hype cycle to development of nematode and particularly banana Xanthomonas wilt resistance is a shallower evidence base provided by a much smaller number of researchers involved in than those working on Bt. Despite this limitation, the analysis does suggest that nematode resistance technologies are within 5–10 years of achieving the plateau of productivity based on the similar stages of development in Bt insect resistance. A similar timeline is likely for the banana Xanthomonas wilt resistance technologies that are at a similar stage of development to the nematode resistance technologies.

The most substantial issue for the development of these public good technologies is maintaining a level of donor support required for the translation phases, by comparison the development of Bt in cotton and maize was more assured due to investment by biotechnology companies. Science‐related factors include the need to demonstrate efficacy across all African regions where marketing is anticipated. Resistance breaking might eventually require management but is more likely to limit the duration of productivity rather emerge before widespread uptake. A more substantial issue is the capacity within Africa to produce the many millions of transgenic plantlets that would be required. Data compiled to date independently of the technology developers establish that neither of the new banana technologies poses toxicological or allergenic risk. Consequently, regulatory actions that resulted in withdrawal of soybeans expressing a novel protein that proved to be an allergen (Herman 2003) seem unlikely. However, regulatory processes have not yet optimized in Africa to support rapid and safe uptake of beneficial crops (Atkinson et al. 2015). A further major issue is political concerns that have hindered progress for aubergine (Brinjal) in India but not Bangladesh (Herring 2015). The future scientific objectives in addition to translational effort is to increase the benefits offered by transgenic banana by stacking traits such as resistance to nematode and banana Xanthomonas wilt in grower‐preferred cultivars.

## Conflict of Interest

The authors declare no competing financial interests.
